# Prevalence and incidence of major depressive disorders among people living with HIV residing in Africa: a systematic review and meta-analysis protocol

**DOI:** 10.1186/s13643-018-0672-2

**Published:** 2018-01-12

**Authors:** Jean Joel Bigna, Lewis N. Um, Serra Lem Asangbeh, Aurelie T. Sibetcheu, Arnaud D. Kazé, Jobert Richie Nansseu

**Affiliations:** 1 Department of Epidemiology and Public Health, Centre Pasteur of Cameroon, PO Box: 1274, Yaoundé, Cameroon; 2 School of Public Health, Faculty of Medicine, University of Paris Sud XI, Le Kremlin-Bicêtre, France; 3 Faculty of Medicine and Biomedical Sciences, University of Yaoundé 1, Yaoundé, Cameroon; 4 Agence Nationale de Recherche sur le Sida et les hépatites virales, Yaoundé, Cameroon; 5 Department of Pediatrics, Faculty of Medicine and Biomedical Sciences, University of Yaoundé 1, Yaoundé, Cameroon; 6 Division of Renal Medicine, Brigham and Women’s Hospital, Harvard Medical School, Boston, MA USA; 7 Department of Public Health, Faculty of Medicine and Biomedical Sciences, University of Yaoundé 1, Yaoundé, Cameroon; 8 Department of Disease, Epidemics and Pandemics Control, Ministry of Public Health, Yaoundé, Cameroon

**Keywords:** Depression, HIV, AIDS, Major depressive disorders, Epidemiology, Prevalence, Incidence, Risk factors

## Abstract

**Background:**

Depression represents one of the most frequent neuro-psychiatric diseases; it seems to be more prevalent in people living with HIV compared to the general population. However, summarized data in the African setting on the topic are scarce. This systematic review and meta-analysis aims at assessing the prevalence and incidence of major depressive disorders (MDD) in HIV-infected African populations residing in Africa.

**Methods and design:**

This review will include observational studies conducted among HIV-infected people residing in Africa, which have reported either the prevalence or incidence of MDD or enough data for its appraisal. Relevant records will be searched using PubMed/Medline, EMBASE, African Journals Online, and Africa Index Medicus. In addition, reference lists of eligible papers and relevant review articles will be screened. Published studies from inception Jan 1, 2000 to Dec 31, 2017 will be considered regardless of language of publication. Two review authors will independently screen, select studies, and extract data, with discrepancies resolved by consensus or arbitration by a third review author. Methodological quality of included studies will be assessed using the scale developed by Hoy and colleagues. Funnel-plots and Egger’s test will be used to determine publication bias. The study-specific estimates will be pooled through a random-effects meta-analysis model to obtain an overall summary estimate. The heterogeneity will be evaluated by the χ^2^ test on Cochrane’s Q statistic. Results will be presented by geographical region and antiretroviral therapy status.

**Discussion:**

This study is based on published data; therefore, ethical approval is not a requirement. The final report of this study in the form of a scientific paper will be published in a peer-reviewed journal and presented at scientific conferences. This review will help to have an overview of the burden of MDD among HIV-infected people residing in Africa.

**Systematic review registration:**

PROSPERO, CRD42017058118.

**Electronic supplementary material:**

The online version of this article (10.1186/s13643-018-0672-2) contains supplementary material, which is available to authorized users.

## Background

The HIV pandemic is to-date one of the major global public health concerns. Globally, there were 36.7 million people living with HIV in 2016; the disease has caused 35 million deaths since the beginning of the epidemic [1]. The vast majority of people living with HIV are in low- and middle-income countries where most African countries are classified [1]. HIV-infected patients often have many complications, including neuro-psychiatric disorders among which depression is one of the most important [2].

Depression is a common mental disorder characterized by sadness, loss of interest or pleasure, feelings of guilt or devaluation of one-self, disturbed sleep or appetite, fatigue and concentration problems [3]. Depression is a common mental disorder that affects more than 350 million people globally. Depression is the leading cause of disability in the world and contributes greatly to the global burden of disease [4]. Diagnosing major depressive disorders (MDD) in the context of HIV is an ongoing challenge for clinicians and researchers, especially in the African context, due to complex biological, psychological, and sociological patterns associated with the HIV disease [5].

Depression in HIV-infected people are caused by a combination of several determinants including biological factors (alterations in the white matter structure, hypothalamic-pituitary-thyroid dysfunction, Tat-protein-induced depressive behavior); psychosocial factors (HIV stigma, occupational disability, body image changes, isolation and debilitation); history or comorbidity of psychiatric illness; and the perinatal period in HIV-infected women [6]. Depression can also negatively impact normal functioning of lymphocytes and alter natural killer cell activity, contributing to the increased mortality in this population [6]. There is body of evidence demonstrating the importance of increasing the detection of MDD and applying an adequate treatment in HIV-infected people [6–8]. While the African continent continues to carry out the greatest burden of HIV infection globally [9, 10], systematic reviews and meta-analyses summarizing the epidemiology of MDD in HIV-infected populations are inexistent. HIV infection can lead to depression and conversely, the occurrence of depression during HIV infection can have a negative impact on the course of the HIV disease. Given that people with HIV are more likely to have depression [11], it is important to measure the burden of this concern in order to have relevant and effective intervention strategies. To curb the burden of MDD in HIV-infected populations, stakeholders, clinicians, and policy makers at national and regional level in the Africa continent should be informed and have detailed epidemiological data of good quality. Willing to fill this critical gap, we plan to conduct a systematic review and meta-analysis aiming to determine the prevalence and incidence of MDD in the HIV-infected population residing inside Africa.

## Review questions

1. What is the prevalence and incidence of MDD in HIV-infected population residing in Africa?
2. What are factors associated with the variation of MDD prevalence in HIV-infected population residing in Africa?

## Methods and design

### Design and reporting

The Centre for Reviews and Dissemination guidelines will be used for the methodology of this review [12]. The Preferred Reporting Items for Systematic Review and Meta-Analysis (PRISMA) guidelines will serve as the template for reporting the present review [13]. For the present protocol, the PRISMA statement for Protocols (PRISMA-P) was used for its reporting (see online Additional file 1) [14]. This review is registered in the PROSPERO International Prospective Register of systematic reviews, registration number CRD42017058118.

### Criteria for considering studies for this review

#### Inclusion criteria

1. Type of studies: observational studies (cross-sectional, case-control, or cohort studies).
2. Type of participants: HIV-infected people residing in the African continent.
3. Type of outcome: prevalence and incidence (or studies giving enough data to compute these estimates if not directly calculated) of MDD as defined in the Diagnostic and Statistical Manual of Mental Disorders V/IV-TR or International Statistical Classification of Diseases and Related Health Problems-10 will be included [15, 16].
4. Outcome measurement: studies in which a validated/standardized measurement tool was used to screen for and diagnose MDD.

#### Exclusion criteria

1. Type of studies: case reports, case series (less than 30 patients), letters, reviews, policy reports, commentaries, and editorials.
2. Types of participants: studies in subgroups of participants selected on the basis of the presence of MDD. Studies conducted in pregnant and breastfeeding women and children will also be excluded.
3. Duplicate reports: the less comprehensive/complete and up-to-date version will be excluded for this review.

### Search strategy for identifying relevant studies

#### Bibliographic database search

A comprehensive and exhaustive search of Medline through PubMed, Excerpta Medica Database, African Journals Online and Africa Index Medicus will be performed to identify all relevant articles published on MDD among HIV-infected people from January 1, 2000 to December 31, 2017 regardless of language of publication. A search strategy based on the combination of relevant terms will be conceived and applied. Both text words and medical subject heading terms will be used. The following terms and their variants will be used for depression: “depression”, “major depressive disorder”, and “melancholia”. For HIV, we will use the terms “HIV”, “AIDS”, and “antiretroviral therapy”. Individual country names for the 54 African countries and African sub-region names such as “West Africa” or “Eastern Africa” will also be used as additional key search terms for more abstracts on the subject. African country names will be introduced both in English and languages relevant to each country for example, “Ivory Coast” and “Côte d’Ivoire”. Where country names have changed over time, old and new names will be included, such as “Zaire” and “Democratic Republic of Congo”. Abstracts of all eligible papers will be reviewed and full texts of articles will be accessed through PubMed, Google Scholar, HINARI or journals’ websites. The main search strategy conducted in PubMed/Medline is shown in Additional file 2. This search strategy will be adapted for searching other database.

#### Searching other sources

A manual search which consists in scanning the reference lists of eligible papers and other relevant review articles will be conducted.

### Selection of studies for inclusion in the review

Studies selection will be managed using EndNote X7. Two review authors will independently identify articles and sequentially screen their titles and abstracts for eligibility. Full-texts of articles deemed potentially eligible will be retrieved. Further, these review authors will independently assess the full-text of each study for eligibility, and consensually retain studies to be included. Disagreements when existing will be solved by a third review author. A screening guide will be used to ensure that the selection criteria are reliably applied by all review authors. Eligible studies in other languages than English or French will be translated using Google Translate and considered for inclusion.

### Data extraction and management

Data will be extracted using a preconceived data abstraction form. Two investigators will independently extract data. These will include:

1. Author details: name of first author and publication year.
2. Study characteristics: country, study design (cross sectional, case-control, cohort), setting (urban and/or rural), sampling method (random vs non-random), data collection period, response rate, and timing of data collection (prospectively vs retrospectively). World Health Organization region in Africa (central, eastern, northern, southern and western Africa) will be derived from country name and therefore assigned to the study.
3. Participants’ characteristics: selection criteria, age (mean, median, range), gender (proportion of males), HIV related data (CD4 count, viral load, type of HIV virus, time since diagnosis, severity of the disease classified as recommended by WHO [17], antiretroviral therapy regimens and duration of treatment).
4. MDD characteristics: diagnostic criteria used/measurement tool used, prevalence/incidence, number of participants tested and diagnosed with MDD overall and by subgroup of interest like gender, ART regimen and status. Where only primary data (sample size and number of outcomes) will be provided, these data will be used to calculate the prevalence or incidence rate estimates. Disagreements between investigators will be reconciled through discussion and consensus, or arbitration by a third investigator whenever necessary. In case of multinational studies, results will be separated to show the estimate within individual countries. When it will not be possible to disaggregate the data by country, the study will be presented as one and the countries in which the study was done will be shown.

### Appraisal of methodological quality of included studies and risk of bias

Included studies will be evaluated for methodological quality using the 10-item rating scale developed Hoy et al. (see online Additional file 3) [18]. Each item will be assigned a score of 1 (Yes) or 0 (No), and each score will be summed across items to generate an overall study quality score. The total score will range from 0 to 10 with the overall score categorized as follows: 8–10: “low risk of bias”, 6–7: “moderate risk”, and 0–5: “high risk”. We intend to present risk of bias and quality scores in a table.

### Data synthesis including assessment of heterogeneity

Data will be analyzed using Stata software (Stata Corp V.14, Texas, USA). Unadjusted prevalence/incidence and standard errors of MDD will be recalculated based on the information of crude numerators and denominators provided by individual studies. Only studies using the same tool for detection of MDD will be pooled in the meta-analysis. To keep the effect of studies with extremely small or extremely large prevalence estimates on the overall estimate to a minimum, the variance of the study-specific prevalence/incidence will be stabilized with the Freeman-Tukey single arc-sine transformation before pooling the data within a random-effects meta-analysis model [19]. Heterogeneity will be evaluated by the Chi-squared test on Cochrane’s Q statistic [20], which will be quantified by I-squared values, assuming that I-squared values of 25, 50, and 75% being representative of low, medium, and high heterogeneity, respectively [21]. When substantial heterogeneity will be detected, we will perform meta-regression and subgroup analyses to investigate the possible sources of heterogeneity using the following grouping variables: mean or median age, sex (female, male), study setting (rural, semi-urban, urban), geographical area (Northern, Central, Western, Eastern, Southern Africa), countries, ART (yes, no), MDD diagnosis criteria (tools used for the measurement), and administration of the questionnaire (auto-evaluation, hetero-evaluation). We will conduct a sensitive analysis including only studies with low risk of bias. We will assess inter-rater agreement between investigators for study inclusion, data extraction, and methodological quality assessment using the Cohen’s Kappa coefficient [22]. If the included studies differ significantly in design, settings, outcome measures, or otherwise, a narrative format will be used to summarize them.

### Assessment of reporting biases

Symmetry of funnel plots and Egger’s test will serve to assess the presence of publication and selective reporting bias [23]. A *p* value < 0.10 will be considered indicative of statistically significant publication bias. In addition, we will perform a trim-and-fill adjusted analysis. This help to perform the Duval and Tweedie nonparametric “trim and fill” method of accounting for publication bias in meta-analysis and estimates the number and outcomes of missing studies, and adjusts the meta-analysis to incorporate the theoretical missing studies [24].

## Discussion

HIV-infected people are more likely to be diagnosed with MDD than the general population [11]. In this population, symptoms of depression are known to be associated with poor adherence to antiretroviral treatment and HIV disease acceleration. In this context, we plan to conduct this review with the aim of estimating the burden of MDD in this vulnerable population. For this review, we also recognized that there may be a high heterogeneity for the prevalence and incidence estimate due to cultural and ethnic diversity in Africa. There may be under-representation of some countries due to variability in the distribution of resources for research, as has been demonstrated in other systematic reviews in Africa [25, 26]. We hope that this review will serve to draw attention and raise awareness on this growing concern in African settings. The current study is based on published data, and as such, ethical approval is not a requirement. The final report of the systematic review, in the form of a scientific paper, will be published in a peer-reviewed journal. Further, findings will be presented at conferences and submitted to relevant health authorities. We also plan to update the review in the future to monitor changes and guide health service and policy solutions.

## Review status

Formal screening of search results against eligibility criteria.

## Additional files

Additional file 1: Preferred Reporting Items for Systematic review and Meta-Analysis Protocols Checklist. (DOC 88 kb)

Additional file 2: Search strategy in PubMed. (DOCX 17 kb)

Additional file 3: Risk of bias tool. (DOCX 14 kb)

## Abbreviations

AIDS: Acquired immunodeficiency syndrome
ART: Antiretroviral treatment
HIV: Human immunodeficiency virus
MDD: Major depressive disorders
PRISMA: Preferred Reporting Items for Systematic Review and Meta-Analysis
