# Supplementary material for: Prevalence and incidence of major depressive disorders among people living with HIV residing in Africa: a systematic review and meta-analysis protocol
Source: Syst Rev. 2018 Jan 12;7:6. doi: 10.1186/s13643-018-0672-2 (PMC5767019; doi:10.1186/s13643-018-0672-2)
Supplement: Supplementary file 2 — Search strategy in PubMed. (DOCX 17 kb) [file 13643_2018_672_MOESM2_ESM.docx]

**Search strategy in PubMed**

| **Search** | **Area** | **Search terms** |
| --- | --- | --- |
| **#1** | **Depression** | Depression OR “Depressive Disorder” OR “Depressive symptom” OR “Emotional depression” OR “Major depression” OR “major depressive disorder” OR "Depressive neurosis" OR "Depressive neuroses" OR "Depressive syndrome" OR "Neurotic depression" OR “Unipolar depression” |
| **#2** | **HIV** | HIV OR AIDS OR “antiretroviral therapy” OR “antiretroviral treatment” OR HAART OR “highly active antiretroviral therapy” |
| **#3** | **Africa** | (Africa OR Algeria OR Angola OR Benin OR Botswana OR "Burkina Faso" OR Burundi OR Cameroon OR "Canary Islands" OR "Cape Verde" OR "Central African Republic" OR Chad OR Comoros OR Congo OR "Democratic Republic of Congo" OR Djibouti OR Egypt OR "Equatorial Guinea" OR Eritrea OR Ethiopia OR Gabon OR Gambia OR Ghana OR Guinea OR "Guinea Bissau" OR "Ivory Coast" OR "Cote d'Ivoire" OR Jamahiriya OR Kenya OR Lesotho OR Liberia OR Libya OR Madagascar OR Malawi OR Mali OR Mauritania OR Mauritius OR Mayotte OR Morocco OR Mozambique OR Namibia OR Niger OR Nigeria OR Principe OR Reunion OR Rwanda OR "Sao Tome" OR Senegal OR Seychelles OR "Sierra Leone" OR Somalia OR "South Africa" OR "St Helena" OR Sudan OR Swaziland OR Tanzania OR Togo OR Tunisia OR Uganda OR "Western Sahara" OR Zaire OR Zambia OR Zimbabwe OR "Central Africa" OR "Central African" OR "West Africa" OR "West African" OR "Western Africa" OR "Western African" OR "East Africa" OR "East African" OR "Eastern Africa" OR "Eastern African" OR "North Africa" OR "North African" OR "Northern Africa" OR "Northern African" OR "South African" OR "Southern Africa" OR "Southern African" OR "sub Saharan Africa" OR "sub Saharan African" OR "subSaharan Africa" OR "subSaharan African") NOT ("guinea pig" OR "guinea pigs" OR "aspergillus niger") |
| **#4** |  | #1 AND #2 AND #3 |
