# Supplementary material for: Prevalence and incidence of major depressive disorders among people living with HIV residing in Africa: a systematic review and meta-analysis protocol
Source: Syst Rev. 2018 Jan 12;7:6. doi: 10.1186/s13643-018-0672-2 (PMC5767019; doi:10.1186/s13643-018-0672-2)
Supplement: Supplementary file 3 — Risk of bias tool. (DOCX 14 kb) [file 13643_2018_672_MOESM3_ESM.docx]

**Risk of bias assessment tool**

| **Risk of Bias Item** |
| --- |
| ***External Validity*** |
| 1. Was the study target population a close representation of the HIV population in relation to relevant variables? |
| 1. Was the sampling frame a true or close representation of the target population? |
| 1. Was some form of random selection used to select the sample, OR, was a census undertaken? |
| 1. Was the likelihood of non-participation bias minimal? |
| ***Internal Validity*** |
| 1. Were data collected directly from the subjects (as opposed to medical records)? |
| 1. Were acceptable case definition of major depressive disorder used? |
| 1. Was a reliable and accepted diagnosis method for major depressive disorder utilized? |
| 1. Was the same mode of data collection used for all subjects? |
| 1. Was the length of the shortest prevalence period for the parameter of interest appropriate? |
| 1. Were the numerator(s) and denominator(s) for the calculation of the prevalence/incidence of hypertension appropriate? |
